# Co-culture metabolomics: a powerful tool for uncovering host-pathogen phenotypes driving *Burkholderia* infections

**DOI:** 10.1128/msystems.01507-25

**Published:** 2025-12-04

**Authors:** Stephanie L. Bishop

**Affiliations:** 1 Department of Biochemistry, Schulich School of Medicine & Dentistry, Western University 6221, London, Ontario, Canada; CNRS Delegation Alpes, Lyon, Rhone Alpes, France

**Keywords:** infection culture metabolomics, melioidosis, *Burkholderia*, airway epithelial cells, HILIC, LC-MS

## Abstract

Melioidosis, caused by the soil-dwelling pathogen *Burkholderia pseudomallei* (Bt), is a severe respiratory infection with limited treatment options. To investigate the host-pathogen metabolic interplay occurring during these intracellular infections, Hicks et al. built upon an *in vitro* co-culture model they developed with airway epithelial cells and Bt as a surrogate pathogen (D. J. Hicks, N. Aiosa, A. Sinha, O. A. Jaiyesimi, et al., mSystems 10:e00611-25, 2025, https://doi.org/10.1128/msystems.00611-25). Using an untargeted metabolomics approach tailored to central metabolism, they identified several host pathways that were altered during the Bt infection: polyamine biosynthesis, nicotinamide adenine dinucleotide salvage, and the tricarboxylic acid cycle. In addition, they found that several bacterial metabolites, including methylated nucleotide bases, peptidoglycan precursors, and amino acid derivatives, were altered due to Bt infection. These results show that co-culture metabolomics is an effective strategy for identifying host-pathogen metabolic phenotypes resulting from bacterial infections and can uncover new therapeutic strategies to combat melioidosis.

## COMMENTARY

Melioidosis, caused by the Gram-negative soil-dwelling pathogen *Burkholderia pseudomallei*, is a major respiratory infection prevalent in the tropics and parts of the continental United States (1). *B. pseudomallei* has been classified as a potential bioweapon as it can infect humans through multiple routes of entry including inhalation, ingestion, and skin abrasion (2, 3). As *B. pseudomallei* exhibits intrinsic multi-drug resistance and there are currently no FDA-approved vaccines against melioidosis (4, 5), new strategies are needed to prevent and fight these infections. The intracellular lifestyle of *B. pseudomallei* elicits complex metabolic alterations in both the host and pathogen (1). Elucidating the metabolic alterations caused by *B. pseudomallei* infections provides a new route to identify therapeutic targets. An emerging strategy uses *in vitro* co-culture models to perform detailed characterizations of chemical changes in both the host and pathogen during infection. Mammalian cell-culture approaches offer several advantages over animal models, including lower cost, greater experimental control, and fewer ethical implications. This exciting approach shows great promise in uncovering new therapeutic targets and prevention strategies for bacterial infections, including melioidosis.

In recent work, Hicks et al. built upon their established *in vitro* melioidosis infection model using airway epithelial cells (AECs) and *Burkholderia thailandensis* (Bt), a surrogate pathogen that has similar pathogenicity but is safer to handle than *B. pseudomallei* (6, 7). This study placed particular emphasis on investigating how the pathogen utilizes the host’s central metabolism and adapts its own metabolism to enhance intracellular survival and pathogenesis. They used a hypothesis-generating technique called untargeted metabolomics to characterize metabolic changes in the AECs elicited by an intracellular Bt infection. The authors used liquid chromatography-mass spectrometry instrumentation, with a type of chromatography called HILIC, to analyze polar metabolites such as amino acids, organic acids, sugars, and vitamins. This allowed the authors to explore metabolic changes in central metabolic pathways, which are necessary for growth and reproduction. Importantly, the authors used a multi-step approach to annotate (putatively identify) metabolites, which led to high-confidence annotations of metabolites they detected in central metabolic pathways (8). They acquired data in full scan mode to detect the intact molecule and used a fragmentation mode called MS^2^, which generated a “molecular fingerprint” for each molecule. They then matched these MS spectra to spectral libraries and an in-house metabolite standard library to identify candidate metabolites by their exact mass, fragmentation spectrum, and when possible, retention time. This detailed metabolite annotation approach is important for study reproducibility and to ensure that the authors select biologically relevant metabolic pathways as the focus for future studies.

Using this untargeted metabolomics strategy, Hicks et al. compared metabolic profiles of AECs at different multiplicities of infection (number of bacteria relative to the number of host cells) and exposure times (7). The authors used statistical approaches such as principal component analysis and hierarchical clustering analysis to identify the key drivers of metabolic differences in AECs caused by Bt infection. They found that the duration of Bt exposure, rather than the multiplicity of infection, had a larger impact on the global metabolic profile. They also identified several host metabolic pathways that were altered during infection. Their approach avoided several common pitfalls in metabolic pathway analysis (9). They focused only on central metabolic pathways, containing the polar metabolites detected using their HILIC-MS approach, and examined biologically relevant metabolic pathways for each cell type.

Some of the host metabolic pathways that were altered in the Bt-challenged AECs compared to the mock-challenged control included polyamine biosynthesis, nicotinamide adenine dinucleotide (NAD+) salvage, and the tricarboxylic acid (TCA) cycle. The authors proposed that polyamine acetylation by the enzyme SpeG and agmatine production may be mechanisms used by Bt to adapt to the host’s immunological response (10–12). They observed reduced levels of NAD+ and elevated levels of nicotinic acid in Bt-challenged AECs, suggesting that Bt disrupts the host’s NAD+ salvage pathway to use nicotinic acid during its intracellular lifecycle (13). Therefore, disruption of bacterial nicotinamidase (PNC1), which converts nicotinamide to nicotinic acid, may present a new therapeutic target. The authors found evidence that Bt infection depletes TCA cycle intermediates such as citric acid, fumaric acid, and malic acid, and thereby shifts the host’s metabolism to utilize glycolysis for energy production.

In addition, several bacterial metabolites were altered during the Bt infection, including nucleotide bases, peptidoglycan biosynthesis precursors, amino acid derivatives, and specialized metabolites. Several methylated nucleotide bases were elevated during Bt infection, which have previously been reported in several bacterial infection models (14, 15). They also found evidence that Bt depletes host metabolites such as uridine diphosphate N-acetylglucosamine, glutamic acid, and alanine. These metabolites are used by bacteria to produce peptidoglycan, an important component of the bacterial cell wall. Other depleted metabolites in the Bt infection included tryptophan and taurine derivatives, as well as glutathione metabolites. They found that specialized metabolites such as urocanic acid, ornithine lipids, and quorum-sensing metabolites such as HMAQs were elevated during the infection. These findings show that a co-culture metabolomics approach is effective in elucidating both host and pathogen metabolic alterations occurring during infections.

An interesting aspect about this study was that the authors were able to generate specific hypotheses from their untargeted metabolomics data that can now be investigated with targeted experiments. Hicks et al. provided evidence that Bt disrupts host functions such as energy metabolism and amino acid utilization (7). They identified metabolic alterations in several pathways implicated in host defense and immunometabolic crosstalk, such as polyamine biosynthesis and NAD+ salvage. This led the authors to propose new therapeutic targets such as bacterial nicotinamidase (PNC1) and polyamine acetyltransferase (SpeG). Disrupting these bacterial enzymes could limit the intracellular proliferation of *B. pseudomallei*. Ultimately, this co-culture metabolomics strategy will support the development of more sophisticated model systems such as lung-on-a-chip and organoid models, which better mimic the complex multicellular organization of tissues. These model systems can help us uncover new therapeutic targets needed to combat complex bacterial infections including melioidosis.
